# SUPPORT Tools for evidence-informed health Policymaking (STP) 12: Finding and using research evidence about resource use and costs

**DOI:** 10.1186/1478-4505-7-S1-S12

**Published:** 2009-12-16

**Authors:** Andrew D Oxman, Atle Fretheim, John N Lavis, Simon Lewin

**Affiliations:** 1 Norwegian Knowledge Centre for the Health Services, P.O. Box 7004, St. Olavs plass, N-0130 Oslo, Norway; 2 Norwegian Knowledge Centre for the Health Services, P.O. Box 7004, St. Olavs plass, N-0130 Oslo, Norway; Section for International Health, Institute of General Practice and Community Medicine, Faculty of Medicine, University of Oslo, Norway; 3 Centre for Health Economics and Policy Analysis, Department of Clinical Epidemiology and Biostatistics, and Department of Political Science, McMaster University, 1200 Main St. West, HSC-2D3, Hamilton, ON, Canada, L8N 3Z5; 4 Norwegian Knowledge Centre for the Health Services, P.O. Box 7004, St. Olavs plass, N-0130 Oslo, Norway; Health Systems Research Unit, Medical Research Council of South Africa

## Abstract

*This article is part of a series written for people responsible for making decisions about health policies and programmes and for those who support these decision makers*.

In this article, we address considerations about resource use and costs. The consequences of a policy or programme option for resource use differ from other impacts (both in terms of benefits and harms) in several ways. However, considerations of the consequences of options for resource use are similar to considerations related to other impacts in that policymakers and their staff need to identify important impacts on resource use, acquire and appraise the best available evidence regarding those impacts, and ensure that appropriate monetary values have been applied. We suggest four questions that can be considered when assessing resource use and the cost consequences of an option. These are: 1. What are the most important impacts on resource use? 2. What evidence is there for important impacts on resource use? 3. How confident is it possible to be in the evidence for impacts on resource use? 4. Have the impacts on resource use been valued appropriately in terms of their true costs?

## About STP

*This article is part of a series written for people responsible for making decisions about health policies and programmes and for those who support these decision makers. The series is intended to help such people ensure that their decisions are well-informed by the best available research evidence. The SUPPORT tools and the ways in which they can be used are described in more detail in the Introduction to this series *[1]. *A glossary for the entire series is attached to each article (see Additional File *1*). Links to Spanish, Portuguese, French and Chinese translations of this series can be found on the SUPPORT website http://www.support-collaboration.org. Feedback about how to improve the tools in this series is welcome and should be sent to: STP@nokc.no*.

## Scenario

*You work in the Ministry of Health and the Minister of Health has asked you to brief her on the costs of options being considered as part of a healthcare reform programme*.

## Background

In this article, we present four questions that policymakers and those who support them can ask when assessing the costs of a policy or programme option. Such questions could be applied, for instance, in the scenario outlined above. Our focus is on finding and using evidence related to resource use and the costs of a policy or programme option, rather than on cost-effectiveness analysis or other types of economic analysis.

Policymakers want to ensure that policies represent good value for money, as do those affected by them. To do this it is essential to consider the costs of options as well as their health and other impacts. Option costs differ from other impacts in a number of key ways [2]:

• *Healthcare costs are typically shared*. For most impacts other than costs, it is usually clear who will be advantaged and who will be disadvantaged, though this may not be the case for all outcomes. An entire community will benefit from a vaccination programme because of the herd effect (the reduced transmission of the disease once most community members are vaccinated). Similarly, in the case of the widespread use of antibiotics to treat individual infections, downstream adverse consequences of drug resistance may occur for the wider community. These are exceptions for health outcomes. On the other hand, healthcare costs are typically shared by the government, private insurers, employers and patients. And within a society, how costs are shared may differ still further depending on patient age (e.g. whether they are under or over 65) or circumstance (e.g. whether the patient is receiving social assistance).

• *Unit costs tend to vary widely across jurisdictions*. For instance, the cost per unit of drugs is largely unrelated to the actual costs of production but is instead more closely related to marketing decisions and national policies. Thus, for example, most medicines under patent cost substantially more in the United States than in Canada [3]. Further, costs may vary widely even within jurisdictions. Hospitals or health maintenance organisations may be able to negotiate special arrangements with pharmaceutical companies for substantially lower prices than those available to patients or other providers. Unit costs also vary over time due to inflation, but may vary over time due to factors relating to demand, too (e.g. when a drug is indicated for use in an increased range of clinical applications), and supply (e.g. when a drug comes off patent).

• *Resource use is likely to vary across jurisdictions*. In addition to unit costs, the amount of resources used may vary. This is due to a range of factors, including professional practices (e.g. the extent to which a diagnostic test is requested by clinicians for a particular health problem), service settings (e.g. the balance between primary and secondary care), levels of patient adherence, and reimbursement policies.

• *Resource implications vary widely across jurisdictions*. Even when resource use remains constant, resource implications may vary widely across jurisdictions. A year's supply of a very expensive drug may pay one nurse's salary in the United States, six nurses' salaries in Eastern Europe, and 30 nurses' salaries in Africa. What one can buy with the resources saved if one foregoes the purchase of a drug, vaccine or procedure - and the health benefits achievable with those expenditures - may thus differ significantly [4].

• *Stakeholders have different perspectives regarding the budgetary envelope in which they are considering resource implications*. Individual patients may only be interested in their out-of-pocket costs or may have varying views about risk sharing or who should bear the costs of healthcare. Hospital or district managers who are operating within fixed budgets may consider the cost of an option relative to other possible uses for the same money. Or they may examine the opportunities available to them to shift resources from one use to another. Similarly, a Minister of Health may be interested primarily in healthcare costs and the healthcare budget. Other policymakers, such as those in a Ministry of Finance, may apply a broader perspective and consider the overall government budget, including non-healthcare expenditures and tax increases or reductions.

• *Conflicting interests related to costs are common*. For example, the economic interests of health professionals or industry executives (who typically want to earn as much as possible) may often be in conflict with the interests of society or governments (which typically want to get as much as they can for as little as possible).

**Figure 1:**
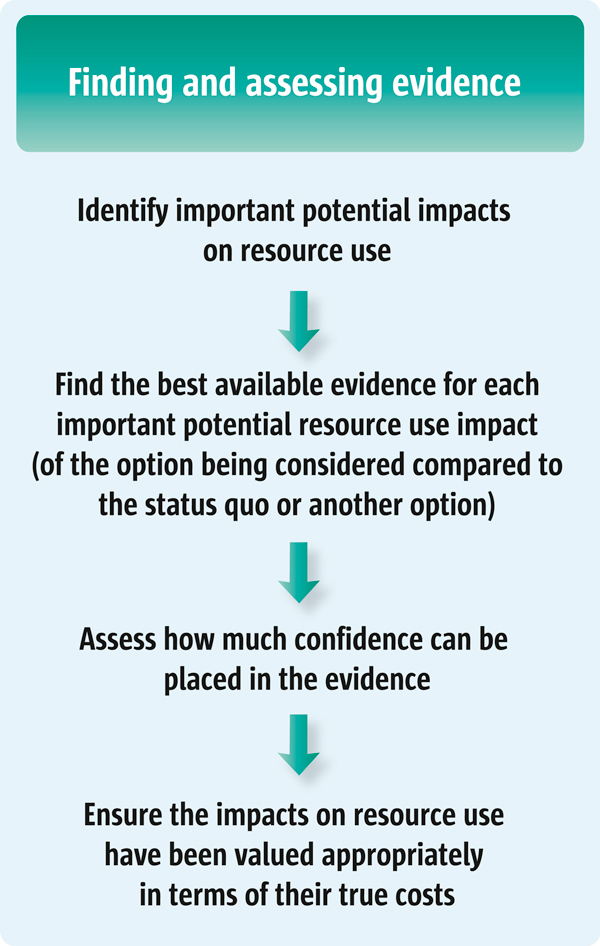
**Four steps necessary to identify and incorporate evidence of the costs of options**.

Despite these differences, cost considerations are similar in many ways to considerations related to other consequences. This is because policymakers and their staff also need to identify important impacts on resource use, and acquire and appraise the best available evidence regarding those consequences to ensure that the resource consequences have been valued appropriately [5-7]. Due to differences between costs and other consequences, a consideration of costs presents special challenges [2,8]. Figure 1 shows four steps that are necessary to identify and incorporate evidence about resource use and costs when considering policy and programme options.

## Questions to consider

The following questions can be used to guide assessments of the costs of potential options:

1. What are the most important impacts on resource use?

2. What evidence is there for important impacts on resource use?

3. How confident is it possible to be in the evidence for impacts on resource use?

4. Have the impacts on resource use been valued appropriately in terms of their true costs?

### 1. What are the most important impacts on resource use?

Health policies and programmes entail the use of resources, particularly human resources such as time. When considering which potential impacts on resource use are important, policymakers should first focus on resource *use *rather than *costs *(see Table 1, for example). Examples of potentially important resource consequences that should be considered include changes in the use of healthcare resources, non-healthcare resources, and patient and informal caregiver time (these and others examples are outlined in Table 2).

**Table 1:** Example: Identifying potentially important resource consequences for a national programme of outreach visits to improve prescribing for hypertension

Systematic reviews have found that educational outreach visits (i.e. personal visits to healthcare professionals in their own settings by trained outreach visitors) have relatively consistent and small, but potentially worthwhile, effects on prescribing [25]. In a randomised trial in Norway, these visits were found to increase the use of thiazides, in adherence with clinical practice guidelines, from 11% to 17% among patients with newly diagnosed hypertension [26]. To determine whether this improvement was worthwhile (in relation to the cost of a national outreach programme), the following uses of resources were considered [27]:	
• Development of software (used to audit medical records and provide feedback to physicians)	
• Training outreach visitors (pharmacists)	
• Printed materials	
• Travel for the pharmacists doing the outreach visits	
• Pharmacists' time	
• Administrative time (e.g. making appointments for the outreach visits)	
• Physicians' time (for the outreach visits)	
• Technical support	
• Drug expenditure	
• Patient visits	
• Laboratory tests	

**Table 2:** Examples of potentially important resource consequences*

1. Changes in use of healthcare resources	
• Policy or programme delivery	
- Human resources/time	
- Consumable supplies	
- Land, buildings, equipment	
• Additional (or fewer) hospitalisations, outpatient visits or home visits	
• Additional (or less) use of laboratory tests or examinations	
• Paid transportation (e.g. emergency transportation)	

**2. Changes in use of non-healthcare resources**	

• Transportation to healthcare facilities	
• Special diets	
• Social services (e.g. housing, home assistance, occupational training)	
• Home adaptation	
• Crime (such as theft, fraud, violence, police investigation, court costs), for example, in relation to options targeted at drug or alcohol abuse	

**3. Changes in use of patient and informal caregiver time**	

• Outpatient visits	
• Hospital admissions	
• Time of family or other informal caregivers	

**4. Changes in productivity**	

• We suggest that changes in productivity and the intrinsic value of changes in health status should be captured in terms of the value or importance attached to health outcomes and should not be included as resource consequences	

* Adapted from Luce and colleagues [10]

When considering which impacts on resource use are important it is essential to consider both the resources used to implement the option (i.e. resource inputs such as drugs, equipment and care) *and *subsequent resource use arising from the impacts of the option on health or other outcomes (e.g. increases or decreases in healthcare utilisation due to the impacts of the option). Incentives to patients to improve adherence to tuberculosis treatment, for example, require substantial resource inputs. These may be offset by subsequent savings if there is a reduction in failed treatment and less spread of the disease (and therefore less subsequent resource use for retreatment and the treatment of others who become infected).

Changes in the productivity of patients may also be important. People with AIDS, for instance, may place a high value on being able to work and earn money, but the process of measuring and valuing actual changes in productivity is controversial [9]. Like others [2,8,10], we suggest that such changes in productivity should be considered as components of the intrinsic value of changes in health status, and should not be included as resource consequences.

On the other hand, some outcomes such as hospitalisations or days in hospital can be considered as important in their own right and also as a component of resource use.

When deciding which resource consequences are potentially important it is necessary to specify the viewpoint from which recommendations are made. One option is to adopt a societal perspective: this is a broad viewpoint that includes all important healthcare and non-healthcare resources [2]. This option has the advantage of ensuring that the issue of who pays does not determine whether resource use is included.

Policymakers may sometimes have a remit to make decisions about the use of resources within a healthcare system. In such instances, costs or savings outside of the healthcare system would not be included. This exclusion would not preclude a consideration of the impacts of an option on issues such as social services or crime, in addition to health outcomes. But any costs or savings associated with those impacts would not be relevant to the healthcare budget unless there was a transfer of funds (e.g. from criminal justice to health).

It is also necessary to specify the time horizon for a policy decision (i.e. the period of time for which resource use, as well as health outcomes and other impacts, will be considered).

### 2. What evidence is there for important impacts on resource use?

Evidence must be found for each potentially important resource consequence. Further, an estimate must be provided of the difference in resource use between implementing the policy or programme on one hand, and the comparator (typically the status quo) on the other (see Table 3 for examples of resources and data sources used in finding evidence of resource consequences). As with health outcomes and other impacts, a comparison is needed regardless of whether it is made implicitly or explicitly. For instance, when considering the option of scaling up the use of artemisinin combination therapy (ACT) for uncomplicated falciparum malaria, increased expenditures on ACT (and corresponding changes in the use of other anti-malarials) must be compared to current expenditures on ACT and other anti-malarials (the status quo). Other resource consequences of scaling up the use of ACT, such as training or providing incentives to community health workers to deliver ACT must also be compared to the status quo (which may vary from setting to setting). Similarly, any subsequent savings resulting from scaling up the use of ACT (e.g. fewer hospitalisations) must also be compared to the status quo. If two competing options for scaling up the use of ACT are being considered, it will be necessary to compare the resource consequences of *both *of these to each other (either directly or indirectly).

**Table 3:** Example: Finding evidence for resource consequences. The following data sources were used to estimate the differences in resource use between a programme of outreach visits (targeted at all general practitioners in Norway) and no programme (the status quo) [27]. The programme is described further in Table 1.

Resources	Data sources
Development of software	Invoices, estimates of time spent
Training of outreach visitors	Estimate of time spent; invoices
Printed materials	Invoice
Travel	Record of travel days, estimate of travel distances
Pharmacists' time	Record of number of visits and days spent on visits
Administrative time	Records and estimates of time expenditure
Physicians' time	Record of length of outreach visit and number of physicians present
Technical support	Records of invoices
Drug expenditure	Medical records
Patient visits	Medical records
Laboratory tests	Medical records

Because data were only collected for one year and from 139 practices (501 physicians, half of whom received outreach visits and half of whom did not) it was necessary to extrapolate the use of resources beyond one year and to the rest of the country.

Systematic reviews, randomised trials and observational studies may provide evidence of the impacts of options on resource use. Such evidence can be published in, or separately from, clinical studies or impact evaluations. The use of resources in specific settings can be retrieved from national or local databases, such as prescription databases for drug use, and hospital databases for information related to hospitalisations [11].

Evidence of resource use may also come from sources other than those used to obtain evidence of health benefits. This may be the case because:

• Trials or impact evaluations (and systematic reviews of these) do not fully report resource use

• Trials and impact evaluations may not fully reflect the circumstances - and thus the resource use - in the setting where a policy decision must be made, and

• The relevant resource use may extend beyond the duration of the trial or impact evaluation

Evidence of resource use should be in natural units, such as visits, hospitalisations or the number of doses of ACT. There are two reasons for this. Firstly, when only total costs are reported (i.e. the number of units of a resource multiplied by the unit cost of the resource), resource use cannot be separated from unit costs, which might vary considerably between settings and over time. Secondly, without information about resource use it is difficult to make judgements about the validity and the applicability of the evidence.

Unfortunately, studies sometimes report costs but do not report the underlying levels of resource use. This was apparent in an economic evaluation of magnesium sulphate for pre-eclampsia which reported the total cost, but not the resource use for magnesium sulphate, or the resources for administering magnesium sulphate and other hospital resources [12]. Differences in costs could be due to differences in underlying levels of resource use, differences in unit costs, or both.

Often it is not possible to find evidence for components of resource use that are important for policy decisions. A guideline panel convened by WHO to develop recommendations for the prevention of postpartum haemorrhage, for instance, found very limited evidence of resource use for oral misoprostol compared to intramuscular oxytocin [8]. The panel considered hospitalisation, personnel time, and drugs to be potentially important resource consequences but did not find any evidence for the first two types of resources. The resource consequences of these two options for preventing postpartum haemorrhage were therefore very uncertain.

### 3. How confident is it possible to be in the evidence for impacts on resource use?

The quality of evidence for resource use must be assessed for each important resource consequence (see Table 4). This is because the quality of evidence may be better for some consequences (e.g. drug use) than for other consequences (e.g. personnel time). The criteria for assessing the quality of evidence for resource use are largely the same as those for health outcomes [2,7,8,13]. These include: assessing the study design and other study limitations (i.e. the risk of bias), the precision of the estimate, the consistency of the results, the directness of the evidence (see below), and the risk of publication bias. Factors that often lower the quality of resource evidence (i.e. those that result in less confidence in estimates of resource consequences) include:

• The unavailability of data due to resource use not having been measured or reported, or reported only as cost estimates (in other words, without the data upon which those estimates were based)

• Weak (observational) study designs

• Indirectness due to uncertainty about the transferability of resource evidence from one setting to another, and

• Indirectness due to inadequate follow-up periods. This makes it necessary to extrapolate beyond the length of available studies in order to estimate resource consequences

**Table 4:** Example: Assessing the quality of evidence for resource consequences. The quality of the evidence for the estimates of difference in resource use between a programme of outreach visits (targeted at all general practitioners in Norway) and no programme (the status quo) varied. (See also Tables 1 and 3.)

Resources	Data sources
Development of software	High quality
Training of outreach visitors	High quality
Printed materials	High quality
Travel	Moderate quality*
Pharmacists' time	Moderate quality*
Administrative time	High quality
Physicians' time	Moderate quality*
Technical support	High quality
Drug expenditure	Moderate to low quality^†^
Patient visits	Moderate to low quality^†^
Laboratory test (potassium)	Moderate to low quality^†^

* The evidence for travel, pharmacists' time and physician time was of moderate quality. This was because of uncertainty about the extrapolation of data from practices in the trial to the rest of the country

^† ^The evidence for drug expenditures, patient visits and laboratory tests was of moderate to low quality. This was because of uncertainty about the extrapolation of data from the trial to the rest of the country and, in addition, because of extrapolation beyond one year (the duration of the trial) to estimate the resource consequences over several years for a programme targeted at all general practitioners in the country

Typically, when estimating the cost-effectiveness of a policy or programme, many assumptions must be made. Economic models that are used to estimate cost-effectiveness are valuable given that they can help to make such assumptions explicit. They also allow for sensitivity analyses that test how robust estimates of cost-effectiveness are in relation to those assumptions. It should be noted however, that the various checklists used to assess the quality of economic analyses in the healthcare literature are not constructed to assess the quality of the evidence upon which the analyses were based [14]. Rather, these checklists tend to focus on the quality of the reporting.

Moreover, although published cost-effectiveness analyses can be helpful, particularly for developing a model, they are often of limited value to policymakers when they are not from a policymaker's own setting. The assumptions made and the unit costs that were used may not be transferable from the setting where the analysis was done to one where a decision must be made. Also, as with any research, cost-effectiveness analyses can be flawed. Without knowledge of the complete model it is difficult to make informed judgements about either the quality of the evidence or its applicability [2,8,15,16].

### 4. Have the impacts on resource use been valued appropriately in terms of their true costs?

Attaching appropriate monetary values to resource use can help policymakers to value resource use consistently and appropriately (see Table 5 for examples of relevant data sources). In principle, these values should reflect opportunity costs - that is, the benefits foregone by diverting the resources from the next best alternative use [17].

**Table 5:** Example: Attaching monetary values to resource consequences. The following data sources were used to estimate the monetary value of differences in resource use between a programme of outreach visits (targeted at all general practitioners in Norway) and no programme (the status quo) [27]. (See also Tables 1, 3 and 4.)

Variable	Data sources for monetary values
Development of software	Invoices, salary payments
Training of outreach visitors	Salary payments
Printed materials	Invoice
Travel	Travel invoices
Pharmacists' time	Salary payments
Administrative time	Salary payments, standard estimates for overheads, office rental figures
Physicians' time	Standard tariff for interdisciplinary meetings
Technical support	Invoices
Drug expenditure	"Felleskatalogen 2003" (a Norwegian list of drugs and prices)
Patient visits	Standard tariff for consultation
Laboratory test (potassium)	Standard tariff

Cost calculations based on reliable databases or data sources in the same jurisdiction are the most reliable sources of data for unit costs [18]. Monetary valuations of resource use should be made with data that are specific to the context where a policy decision must be made using transparent and locally relevant unit costs. If this is not possible, purchasing power parity (PPP), exchange rates and inflation factors could be used to assist interpretation of monetary valuations from other settings or times [19]. In a study estimating the cost of cervical cancer screening in five developing countries [20], for example, unit cost data were derived from more than one year. Country-specific deflators were therefore used to adjust all costs to the same price year. Further, to aid cross-country comparability, PPP exchange rates were used to convert costs expressed in local currency units to dollars. Both were measured according to the relevant values in the price year 2000.

Discounting is used in economic evaluations to adjust for social or individual preferences over the timing of costs and health benefits. This means that less weight is given to costs or benefits occurring further in the future than those expected imminently. Recommended discount rates differ between countries and are often varied in sensitivity analyses.

When costs are presented, these should be reported using the appropriate discount rate for the context where the policy decision must be made. Data used to calculate the discounted costs - including quantities of all resource items, unit costs, and the discount rate - should be transparent so that it is possible to assess the validity and applicability or appropriateness of each component.

## Conclusion

**Figure 2:**
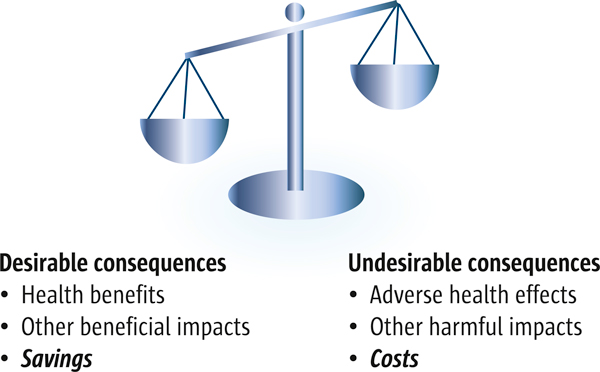
**Balancing the pros and cons of health policies and programmes, including resource consequences**. Resource consequences (the savings or costs of a policy or programme compared to the status quo or other alternative) need to be considered along with health and other impacts when making judgements about the balance between the pros and cons of health policies and programmes

Policymakers and others are concerned with getting value for money; in other words, that health policies and programmes are cost-effective (efficient). Evidence of resource use and costs is needed to inform judgements about cost-effectiveness. We discuss making judgements about the balance between the pros and cons (including savings and costs) of policies and programmes (as illustrated in Figure 2) in a later article in this series [21].

Evidence of resource use and costs is also needed to inform judgements about equity [22]. In addition to considering the overall costs (and cost-effectiveness) of policies and programmes, policymakers need to consider who will bear particular costs and the impact that this will have on inequities.

In terms of both efficiency and equity it is important to ensure that all potentially important resource consequences are identified. It is also essential that the best available evidence is used, and that important uncertainties about resource (and other) consequences are acknowledged and addressed [23,24].

## Resources

### Useful documents and further reading

- Guyatt GH, Oxman AD, Kunz R, Jaeschke R, Helfand M, Vist GE, Schunemann HJ, and the GRADE Working Group. Incorporating considerations of resource use. BMJ 2008; 336:1170-3.

- Brunetti M, Oxman AD, Pregno S, Lord J, Shemilt I, Vale L, et al. GRADE guidelines: 10. Special challenges - resource use. J Clin Epidemiol. In press.

### Links to websites

- Campbell & Cochrane Economics Methods Group. http://www.c-cemg.org - The Campbell & Cochrane Economic Methods Group is an international network of individuals with an interest and expertise in approaches to evidence synthesis that combine economics and systematic review methods.

- GRADE Working Group. http://www.gradeworkinggroup.org/index.htm - The Grading of Recommendations Assessment, Development and Evaluation (GRADE) Working Group has developed a system for grading the quality of evidence and the strength of healthcare recommendations. The system includes an approach to the grading of resource use evidence and the incorporation of evidence into recommendations.

- International Health Economics Association. http://www.healtheconomics.org - The International Health Economics Association was formed to increase communication among health economists, foster a higher standard of debate in the application of economics to health and healthcare systems, and assist young researchers at the start of their careers.

- Office of Health Economics, United Kingdom. http://www.ohe.org/page/index.cfm - The Office of Health Economics provides independent research, advisory and consultancy services on policy implications and economic issues within the pharmaceutical, healthcare and biotechnology sectors.

- CCEMG - EPPI-Centre Cost Converter. http://eppi.ioe.ac.uk/costconversion/default.aspx - a simple web-based tool that can be used to adjust an estimate of cost expressed in one currency and price year, to a target currency and/or price year.

- NHS Economic Evaluation Database (NHS EED). http://www.crd.york.ac.uk/crdweb/Home.aspx?DB=NHS%20EED&SessionID=&SearchID=&E=0&D=0&H=0&SearchFor= - NHS EED contains 24,000 abstracts of health economics papers including over 7,000 quality-assessed economic evaluations. The database aims to assist decision makers by systematically identifying and describing economic evaluations, appraising their quality, and highlighting their relative strengths and weaknesses.

## Competing interests

The authors declare that they have no competing interests.

## Authors' contributions

ADO prepared the first draft of this article. AF, JNL and SL contributed to drafting and revising it.

## Supplementary Material

Additional file 1 Glossary Click here for file
